# Imperative Role of Machine Learning Algorithm for Detection of Parkinson’s Disease: Review, Challenges and Recommendations

**DOI:** 10.3390/diagnostics12082003

**Published:** 2022-08-19

**Authors:** Arti Rana, Ankur Dumka, Rajesh Singh, Manoj Kumar Panda, Neeraj Priyadarshi, Bhekisipho Twala

**Affiliations:** 1 Computer Science & Engineering, Veer Madho Singh Bhandari Uttarakhand Technical University, Dehradun 248007, Uttarakhand, India; 2 Department of Computer Science and Engineering, Women Institute of Technology, Uttarakhand Technical University (UTU), Dehradun 248007, Uttarakhand, India; 3 Division of Research and Innovation, Uttaranchal Institute of Technology, Uttaranchal University, Dehradun 248007, Uttarakhand, India; 4 Department of Project Management, Universidad Internacional Iberoamericana, Campeche 24560, Mexico; 5 Department of Electrical Engineering, G.B. Pant Institute of Engineering and Technology, Pauri 246194, Uttarakhand, India; 6 Department of Electrical Engineering, JIS College of Engineering, Kolkata 741235, West Bengal, India; 7 Digital Transformation Portfolio, Tshwane University of Technology, Staatsartillerie Rd, Pretoria West, Pretoria 0183, South Africa

**Keywords:** Parkinson’s disease, machine learning, artificial neural network, logistic regression, support vector machine, classification

## Abstract

Parkinson’s disease (PD) is a neurodegenerative disease that affects the neural, behavioral, and physiological systems of the brain. This disease is also known as tremor. The common symptoms of this disease are a slowness of movement known as ‘bradykinesia’, loss of automatic movements, speech/writing changes, and difficulty with walking at early stages. To solve these issues and to enhance the diagnostic process of PD, machine learning (ML) algorithms have been implemented for the categorization of subjective disease and healthy controls (HC) with comparable medical appearances. To provide a far-reaching outline of data modalities and artificial intelligence techniques that have been utilized in the analysis and diagnosis of PD, we conducted a literature analysis of research papers published up until 2022. A total of 112 research papers were included in this study, with an examination of their targets, data sources and different types of datasets, ML algorithms, and associated outcomes. The results showed that ML approaches and new biomarkers have a lot of promise for being used in clinical decision-making, resulting in a more systematic and informed diagnosis of PD. In this study, some major challenges were addressed along with a future recommendation.

## 1. Introduction

The brain of humans is the main computing unit of the human body, and if there is any minor accident in any part of the human body, then it will directly affect the other organs. One of its silent effects is PD [1]. PD is a neurological disease that is incurable and is progressive over time [2]. As of 2020, an estimated 9.4 million people were still living with this disease worldwide [3]. This disease mostly affects people over the age of 60 years, with only 4% of the cases occurring in people under the age of 50 [4]. The symptoms of this disease are featured as motor and non-motor [5]. The main motor symptoms are slowness of movement, tremor, rapid eye movement disorder, shivering, gait issue, and unstable posture [6,7]. Non-motor symptoms include hypotension, sweating in the body, fatigue, constipation, urinary problems, and loss of weight [8]. Several studies that have been conducted by researchers have shown that 90% of PD patients have speech and voice acoustic problems [9], including microphonia, monochromatic, dysarthria, and dysphonia [10]; thus, as a result, the initial symptom observed in patients with this disease is a loss of voice [11]. At present, there is no established treatment for the disease [12]; however, there are a number of pharmacological therapies that can significantly reduce symptoms, particularly in the early stages. The analysis of the frequency of voice is concise and non-invasive. As a result, the frequency of voice can be used to track the progression of this subjective disease [13]. To check the progression of this disease, many speech experiments have been conducted. In the field of the medical (healthcare) sector, ML approaches are being continuously used. ML algorithms are being used on a variety of data modalities, including acoustic voice recording and handwritten patterns for the diagnosis of PD. With the help of ML techniques, we may recognize the appropriate attributes that are not traditionally applied in the medical diagnosis of PD and depend on these alternative indicators to diagnose PD in its preclinical phases. In general, there are three phases to the diagnosis of this disease, (1) pre-processing data, (2) extracting features, and (3) applying classification techniques [14,15,16]. In the very first phase, the categorization of speech signals with time frames is conducted. The filter method is used to remove any noise that may be present. In the second phase, frequently used features are extracted from each segment. Finally, in the last phase, the classification techniques are performed. The approach utilized for feature extraction is heavily influenced by the classification technique’s performance. Hence, choosing the appropriate classification technique is a big issue that needs to be considered for this disease. The review discussed in this work aimed to look at the usage of ML models trained on sensory data to assist PD patients, their careers, and physicians throughout the stage of the treatment. Consequently, it presented the key findings of several research publications that provide PD prediction and estimating models based on cutting-edge IoT technology and ideal sensor installations. The goal was to provide neurologists with important insights that may improve PD diagnosis and treatment by shedding light on further unique techniques and presenting novel solutions that have not been sufficiently covered in the reviews that have already been published. The contribution of this paper is as follows:

- We introduce the background knowledge of Parkinson disease with main characteristics and major motor and non-motor symptoms.
- We classified ML models and also analyzed the accuracy of ML models for the diagnosis of Parkinson disease on the basis of speech, handwriting, and gait parameters.
- In this paper, a different ML-based framework for the diagnosis of Parkinson disease is also discussed, with the objective of enhancing Parkinson disease data.
- Finally, the article highlights the challenges and discusses the recommendations for the future work.

The structure of the study is as follows: Section 2 discusses the methodology of the study; Section 3 illustrates the term PD in detail with the clinical method used to diagnose PD. Section 4 provides the ML techniques used to diagnose PD with the classification of the ML algorithm. Section 5 examines the adaptation of the ML algorithm with the proposed architecture of the voice dataset and a handwriting pattern to diagnose PD. Section 6 illustrates the discussion of challenges and recommendations. Finally, Section 7 defines the conclusion.

## 2. Methodology of the Study

### 2.1. Data Acquisition

In this study, for diagnosis of the subjective disease, the scientometric data were gathered from the Web of Science database, IEEE, ScienceDirect, and Scopus database, with the latest from 2022. These databases have abstract and conceptual data from various research publications. The Scopus database provides a homogenous and standardized search technique, a significant exploration of relevant journals in a variety of diseases, including PD, and the diagnosis of the disease using artificial intelligence techniques. It provides a faster indexing methodology and covers a wide range of publications, making it much easier to access more recent research papers. Utilizing these resources has the additional benefit of delivering excellent multidisciplinary coverage to other well-known databases.

For this review article, a total number of 1432 documents were searched, including the number of book chapters, patents, review articles, implemented research articles, abstracts, and different categories of documents. Out of 1432 results acquired, 1211 were research articles, reviews, book chapters, and patents. All of these records were filtered in order to standardize the data. After filtration, only 50% of the 1211 research articles were obtained. Finally, 112 articles were obtained after limiting the search to only include English-language articles. In order to filter the works, abstracts were examined, and if there was any uncertainty regarding the relevancy of the results, full-length articles were retrieved. The final analyses, which focused on publications on the diagnosis of Parkinson’s disease using ML techniques, were chosen from those articles to ensure the inclusion of pertinent research.

### 2.2. Journals

**Figure 1:**
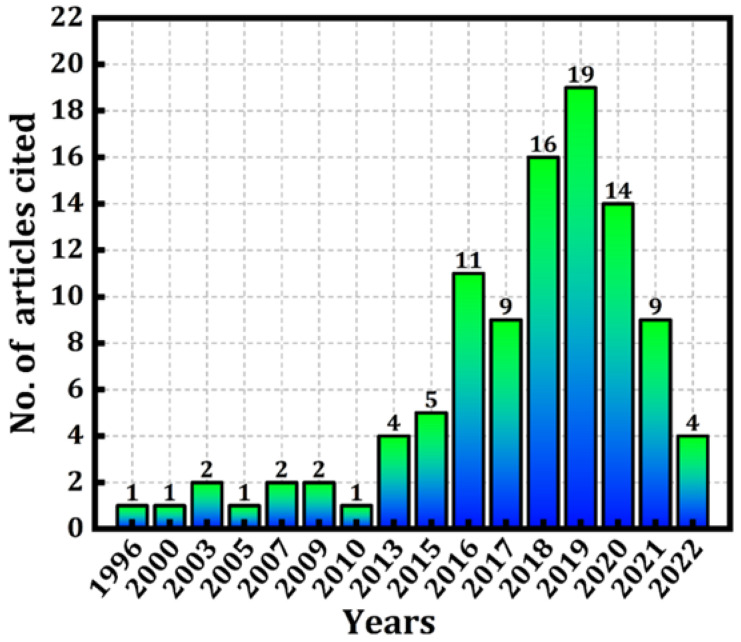
A number of articles cited between 1996 and 2022.

**Figure 2:**
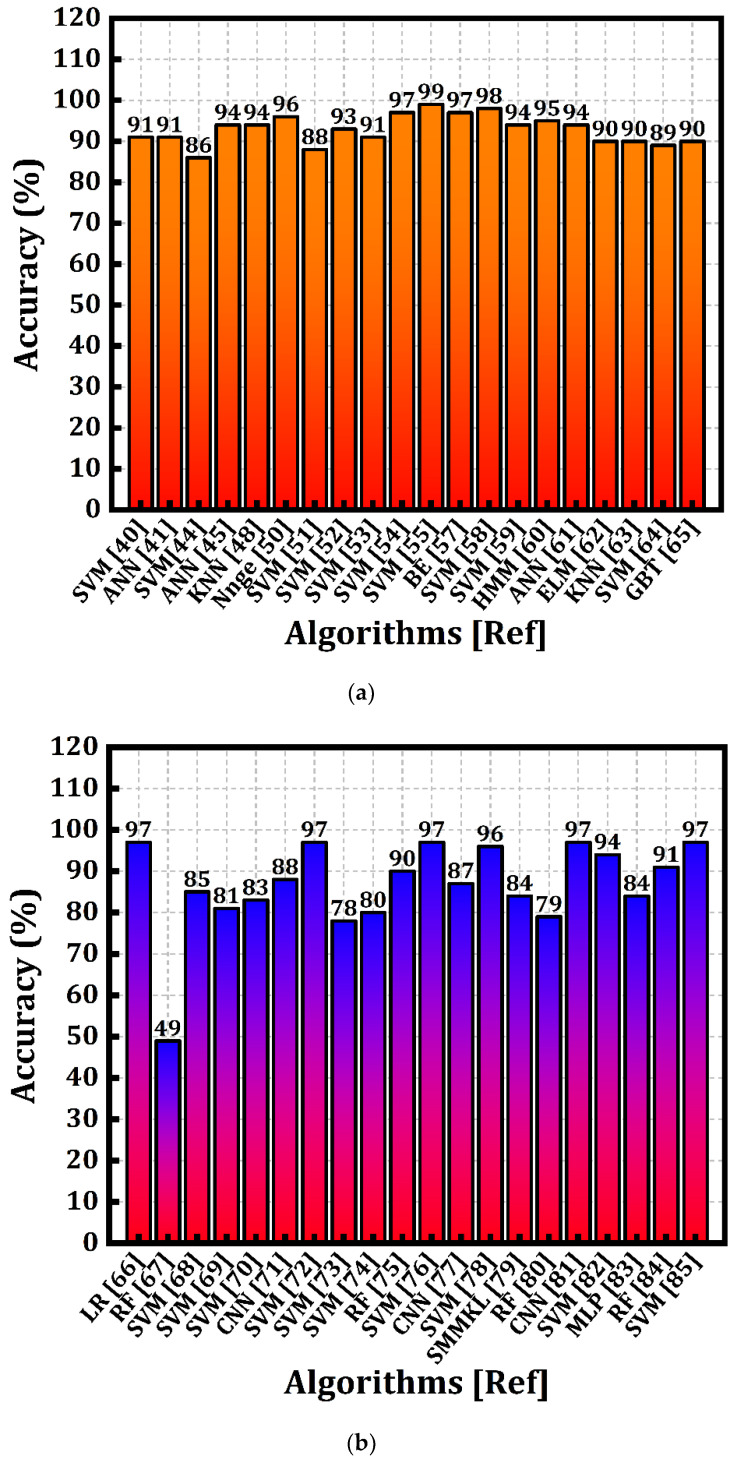
Comparative analysis of machine learning algorithms used to diagnose Parkinson’s disease w.r.t. accuracy rate. (**a**) Accuracy rate of detecting Parkinson’s disease based on speech feature (2015–2020). (**b**) Accuracy rate of detecting Parkinson’s disease based on handwritten pattern features (2015–2020).

A systematic analysis of the diagnosis of PD using the ML approach research was performed by analyzing 103 articles published during 1996–2022. Figure 1 represents the main journal publishing on the diagnosis of PD using various ML techniques. For this current study, we studied the research articles from the Electronics journal of MDPI (8), PubMed (18), IEEE Access (3), IEEE transactions (4), Elsevier (13), and Hindawi (3). The studies considered in this review provide evidence that useful knowledge can be extracted by using feature selection techniques with the help of ML algorithms, regarding motor and non-motor symptoms of PD, allowing doctors to make evidence-based decisions on the available dataset. Figure 2 is the graphical representation of the voice feature and handwritten pattern dataset used to detect PD with an accuracy rate obtained by various algorithms from the years 2015–2020.

## 3. Parkinson’s Disease: Background

PD is a dynamic sensory system problem that influences the development of an individual. Symptoms of PD start gradually and may begin with a hardly detectable tremor [17]. Tremors are common; however, disorders are often accompanied by rigorousness or slowed mobility [18]. In the beginning phases of PD, one’s face might show slight or zero expressions, and one’s arms may not swing when walking. Voice might turn out to be slurred or soft [19]. PD symptoms deteriorate as the disease progresses over time. Although there is no cure for PD, meds could alleviate the symptoms. Doctors may recommend a medical procedure to control specific areas of the brain and further alleviate symptoms. According to research, India has 7 million elderly people suffering from PD [20]. Medication and surgery are offered to control the symptoms of this subjective disease [21]. The number of Americans living with this subjective disease is close to one million, which is greater than the total number of persons with Lou Gehrig’s disease and multiple sclerosis. By 2030, this number is expected to reach 1.2 million.

People with PD (PWP) have significant variances in their symptoms, reactions to medicines, and adverse treatment effects. Understanding the genetic variations among Parkinson’s patients might provide crucial hints regarding how and why each person’s experience with PD differs. Although the actual etiology of PD is unknown, scientists predict that a mix of genetic and environmental factors causes it. Each factor’s impact varies depending on the individual. Researchers do not know why some people develop Parkinson’s and others do not. About 10 to 15% of Parkinson’s cases are genetic in nature. In certain families, variations (or mutations) in particular genes are inherited or handed down from generation to generation [22]. The molecular basis of this neurodegenerative disease is still not fully known over two decades after the discovery of the first mutation linked to PD. Initially, research on the genetics of Parkinson’s disease (PD) concentrated on uncommon family variants of the condition; however, six genes—LRRK2, alpha-synuclein, Parkin, VPS35, DJ-1, and PINK1—have now been conclusively linked to either an autosomal dominant or recessive form of the disease. Major advancements in the field have been made with the introduction of genome-wide association studies (GWAS) and the use of new technologies, such as next-generation sequencing (NGS) and exome sequencing. A wave of genetic association studies later implicated a number of genetic variants in the disease pathogenesis/protection [23].

Proteomic biomarkers, ideally a biomarker that is predicted to properly represent a disease process, should be available for investigation in the afflicted tissue, such as in the suffering dopaminergic neurons in the case of PD. One of the biggest challenges to creating causal or disease-modifying medicines for PD is the fact that this is not achievable. The benefits of this strategy are clear, for instance, in contemporary tumor treatment that may be customized based on the specific hormone receptor status of the malignant cells. Proteomic disease-associated modifications must be looked for inaccessible bodily fluids such as blood plasma or CSF, as well as in peripheral tissues, as this technique cannot be used in PD. Although it initially appears unlikely, there are signs that the observed mutations may in fact represent at least some components of the disease process in the brain [24].

**Figure 3:**
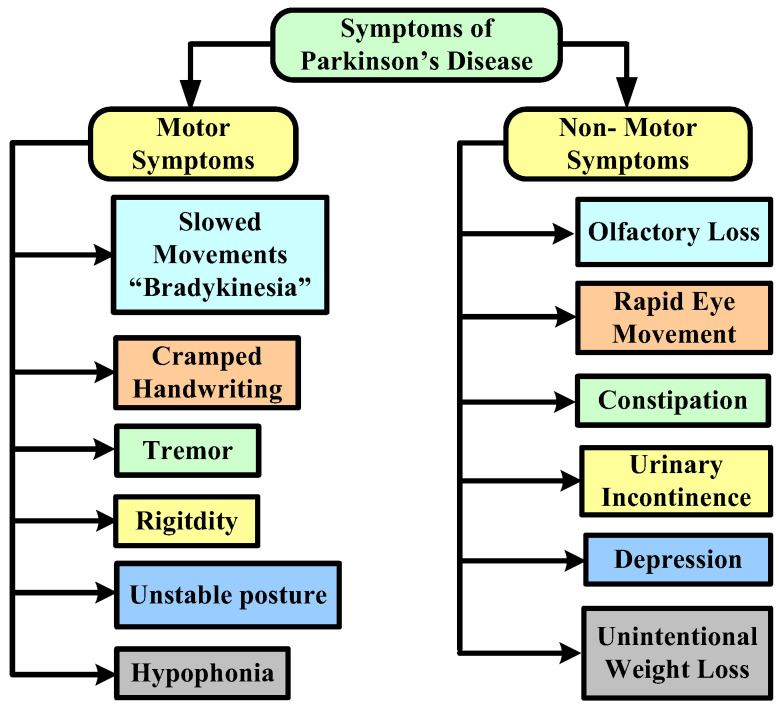
Symptoms of Parkinson’s disease.

In terms of risk factors, although the specific reasons for PD are anonymous, certain cases are frequently caused by natural and other factors that play a significant influence in the progression of the disease. Head traumas, an inadequate diet that includes a large number of pesticides or chemical exposure, and sedentary lifestyles are all risk factors. Figure 3 represents the major symptoms of this disease.

Risk factors for PD include age (this disease rarely affects young adults; it typically manifests in middle or later life, and the risk rises with age), hereditary factors (having close relatives who have the disorder increases your probability of having it), sex (men are more likely to get PD than women), and exposure to toxins (ongoing exposure to herbicides and pesticides may slightly increase risk of PD) [25]. Genetic indicators for PD have been used to identify people who have a higher risk of acquiring this disease, to track the disease’s development, and to look at how well treatment prevents the depletion of dopaminergic neurons. Early diagnostic tools for PD include markers including cerebrospinal fluid testing, non-motor clinical signs of PD, and several imaging modalities [26].

### Clinical Methods Used to Diagnose Parkinson’s Disease

PD is largely diagnosed based on its typical symptoms. The disease cannot be verified by an X-ray or blood test. Although, non-invasive diagnostic imaging, including positron emission tomography (PET), can assist a surgeon in making a diagnosis. Conventional methods for diagnosing Parkinsonism include the presence of two or more primary symptoms, the absence of additional neurological symptoms upon examination, the lack of a history of additional potential causes, such as the use of tranquilizer drugs, head trauma, or stroke, and responsiveness to levodopa or other Parkinson’s medications [27]. Even while the clinical neurological and neuropathological overlap of two or more neurodegenerative disorders (ND) is not unusual, it is still underdiagnosed. The presence or absence of distinctive neuropathologic lesions together with clinical presentation determine the diagnosis of a specific ND. However, in recent years, there has been a growing amount of interest in ND overlaps. To our knowledge, not much research has looked at how often these overlaps are. A single patient may have overlapping NDs due to shared molecular pathogeneses. For instance, both individuals with frontotemporal lobar degeneration (FTLD) and those with amyotrophic lateral sclerosis have the accumulation of misfolded TAR DNA-binding protein-43 (TDP-43) in their bodies (ALS). The idea that diagnostic omission may result in the therapy of one disease while unconsciously allowing the other disease to proceed without intervention is the clinical significance of multiple NDs that overlap with one another. Early detection of overlapping NDs would enable the proper care of each ND before the disease progressed to an advanced state. Clinically recognizing overlapping instances of Lewy body dementia (LBD) is crucial since it can increase morbidity and mortality due to the extreme neuroleptic sensitivity brought on by drugs used to treat other types of dementia. It is also possible for overlapping neurodegenerative disease processes to aggravate symptoms or reduce the threshold of pathology needed for symptoms to appear. For instance, patients who have both Alzheimer’s disease (AD) and LBD may have more severe cognitive or behavioral abnormalities than those who just have AD or LBD. Alternately, for cognitive or behavioral abnormalities to show clinically, a lower threshold of either AD and/or LBD pathogenic changes may be necessary. Although overlapping NDs are not a novel idea, they appear to be underdiagnosed in clinical practice. One of the reasons might be that the symptoms of one ND disguise those of another ND that are occurring concurrently. In neuropathological practice, there may be circumstances when the search for a potential concurrent ND is discontinued after discovering evidence of one ND. Increased awareness and comprehension of the pathophysiology of overlapping NDs might also pave the way for the creation of future treatment plans that might handle numerous NDs together rather than each ND individually [28]. Following are some clinical methods that are used to diagnose PD:

a. Medical Treatment

In most cases, medication is used to treat Parkinson’s patients in order to reduce their disease symptoms. Levodopa drugs or anticholinergic pharmaceuticals stimulate the residual substantia nigra cells to create further dopamine, while levodopa medications suppress part of the acetylcholine production, which restores the homeostasis of the brain’s chemical production. There are a wide variety of side effects associated with each medication class [27]. Levodopa, which was created more than four decades ago, is frequently referred to as the standard of Parkinson’s treatment. Levodopa is used in lower doses in order to reduce symptoms. This development significantly lessens acute vomiting and nausea that are frequently encountered as levodopa side effects. Levodopa often lessens the tremor, stiffness, and slowness symptoms in individuals. Patients with a lack of spontaneous movement and muscular stiffness benefit the most from it [29].

b. COMT Inhibitors

Inhibitors of catechol-O-methyl transferase (COMT) are among the amino groups that contribute to the stability of levodopa levels. Entacapone, tolcapone, and opicapone are the three main COMT inhibitors. These medications work by inhibiting the COMT enzyme, which raises the blood levels of levodopa without causing it to be peripherally degraded into 3-O-methyldopa (3-OMD) [30,31]. Dyskinesia and diarrhea may be possible side effects [27].

c. Anticholinergic medications

The function of the neurotransmitter acetylcholine (ACh) in the central and autonomic nervous systems is blocked by anticholinergic medicines, which leads to a wide range of both beneficial and undesirable consequences. Since many of the most often given medications for seniors are indicated for problems common to the age, one-third to one-half of these medications contain anticholinergic effects [32]. These medications are particularly effective in treating tremors, stiffness of the muscles, and antidepressants Parkinsonism. Due to difficulties and major adverse effects, they are typically not advised for prolonged use in elderly individuals [27].

d. Amantadine

Levodopa-related dyskinesia is typically treated with amantadine as an add-on medication, although more recently, novel long-acting amantadine formulations have been created with additional indications to treat motor fluctuations. Amantadine is hardly associated with impulse control problems and has not been found to produce dyskinesia [33]. Levodopa or anticholinergic medicine may occasionally be used with amantadine. Some of its adverse effects include confusion, sleeplessness, nightmares, irritability, and hallucinations. It may also cause leg swelling [27].

Similar to Parkinson’s disease, Spinocerebellar Ataxia (SCA) can manifest as it, particularly in SCA2, SCA3, and SCA17. SCA2 and SCA17 are more widespread in Asian groups, but SCA3 is more common in western ones. Parkinsonism can occasionally be seen in people with SCA6 and SCA8. The crucial thing to remember is that SCA2 and SCA17 may closely resemble Parkinson’s disease and are a prevalent hereditary cause of Parkinsonism in Asian countries, even in instances that occur sporadically. SCA2, SCA3, and SCA17 screening may thus be necessary in PD patients. The cerebellum and its associated components are impacted by the progressive, autosomal dominant neurodegenerative condition known as spinocerebellar ataxia (SCA). Even while ataxia predominates in the majority of cases, different SCA subtypes exhibit a wide range of clinical traits associated with the brainstem and spinal cord, with and without ataxia. Different SCA subtypes also exhibit a variety of extrapyramidal symptoms, including Parkinsonism. Despite the fact that this patient’s symptoms did not exactly mimic those of idiopathic PD, SCA3 or Machado-Joseph disease (MJD) was the first genetically verified SCA subtype in a patient with the levodopa-responsive Parkinson’s disease (PD) similar phenotype. Many SCA subtypes, including SCA2, SCA6, SCA8, and SCA17, have now been classified as both levodopa-responsive Parkinson’s disease (PD) and typical Parkinsonism [34].

## 4. Machine Learning Techniques Used to Diagnose Parkinson’s Disease

**Figure 4:**
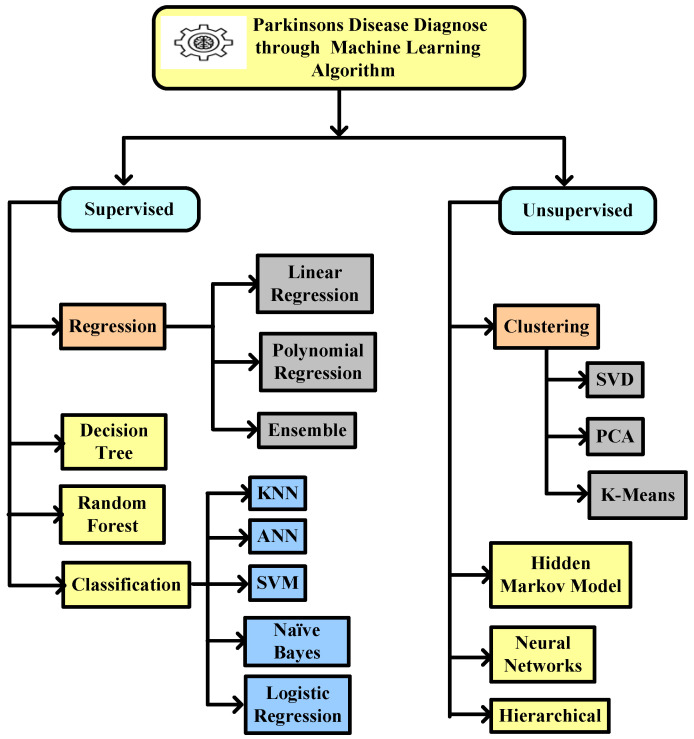
Machine learning algorithm used to diagnose Parkinson’s disease.

This review article identified the potential to correctly estimate the severity of PD, as measured by clinical metrics, using ML techniques such as SVM, ANN, KNN, naïve Bayes, logistic regression, CART, decision tree, etc. ANN is mostly used in classification and regression problems, where existing nearby features are considered to be relatable. Training datasets and test data are used in the ML algorithm. A technique that gains experience from its previous data and improves itself accordingly is known as ML. It is basically an analysis of algorithms that can generate data automatically. An ML classifier is categorized into two types, supervised and unsupervised. Labeled data fall under supervised, where different approaches of algorithms are used to train models. The categorization of ML algorithms is shown in Figure 4. Artificial Neural Network (ANN) [35] and Multilayer perceptron (MLP) with a back propagation algorithm [36] are also used to diagnose PD.

The ML-based diagnosis of this subjective disease can be achieved by using symptoms as an attribute for the algorithm. The ML algorithm is used to diagnose the PD severity from the handwriting of an individual [37]. Speech analysis and tremors are also important risk factors used to diagnose PD [38]. Over time, several initiatives have been taken to diagnose PD by various researchers. The following discussion defines a brief review of major work performed for diagnosing PD from the speech record dataset. In [39], the author discussed a unique methodology to discriminate a healthy person from a person with Parkinson’s disease (PWP) by detecting dysphonia. They introduced a new reliable dysphonia test called pitch period entropy (PPE). It is unaffected by a variety of uncontrolled confounding factors such as loud acoustic surroundings and natural, healthy changes in the voice frequency. The dataset was obtained from thirty-one people, where twenty-three were subjective disease patients and eight were healthy, and it contained 195 persistent vowel phonations. The methodology that was used in this article is categorized into three steps: the calculation of feature refinement, the pre-processing and pre-selection of features, and the classification of models. To diagnose the subjective disease, a kernel support vector machine (SVM) classifier was used. By using this algorithm, the model accomplished an accuracy rate of 91.4%.

The main purpose of this [40] article is to differentiate a healthy person from a PWP. In their work, to create a method for diagnosing PD patients using voice disorders, they used a dataset containing 34 persistent vowels, from 34 individuals, where 17 were subjective patients and 17 were healthy. For classification, the SVM technique was used and achieved an accuracy rate of 91.17% by using the first twelve coefficients of the Mel Frequency Cepstral Coefficients (MFCC) by kernel SVM.

To distinguish healthy individuals from subjective diseases, Ref. [41] used a supervised ML algorithm, SVM, for classification purposes. All the data were processed in a tool called weka. Libsvm was used to find the best plausible accuracy on various kernel values for the given dataset. The linear kernel SVM accomplished an accuracy rate of 65.2174%. Similarly, the poly-kernel and RBF kernel accomplished an accuracy rate of 60.8696%.

In [42], the authors discuss a methodology to diagnose PD. Weka tools were used to develop the algorithms for the pre-processing of data, classification methods, clustering, and the analysis of a given dataset. From the experimental results of this article, the accuracy rate achieved from K-nearest neighbor (KNN) + Adaboost.M1 was 91.28%, KNN + Bagging scores 90.76%, and KNN + MLP score 91.28%.

The author proposed a methodology for distinguishing between healthy persons and subjective disease patients. In their research, the data were obtained from 40 individuals where 20 were healthy and 20 were subjective disease patients [43]. A total of 26 speech samples were taken from each individual, including phrases, sentences, words, and numerals. For classification and cross-validation, they used SVM and KNN. The KNN classifier produced an accuracy of 82.50%, whereas the SVM classifier reported an accuracy of 85%.

In [44], the authors examined several voice signal analysis techniques for the diagnosis of this subjective disease. A novel feature termed tunable Q-factor wavelet transform (TQWT) was presented in their work. TQWT excelled in state-of-the-art voice signal computational methods adopted for feature extraction in PD detection. Distinct classifiers were applied to different feature subsets, and the predictions of the classifiers were aggregated using ensemble methods. The best accuracy of the model was reached by MFCCs and TQWT, which are thus key aspects in the problem of PD classification. As a data preparation phase, the minimum redundancy-maximum relevance (mRMR) feature selection approach was applied. In all the feature subsets, Radial Basis Function (RBF) kernel SVM had the greatest accuracy of 86%.

ANN was used by [45] to identify PD. The dataset was obtained from the University of California, Irvine’s machine learning library. 45 attributes were chosen as input values and one outcome for categorization using the MATLAB tool. With an accuracy of 94.93%, their suggested model was able to differentiate healthy individuals from PD subjects. 

The author addressed the causes and symptoms of the disease. The severity of this disease and its complications were discussed in their work. Furthermore, their studies established the best detection range for classifying Parkinson’s symptoms [46].

The authors discovered a method for diagnosing PD that included ML and Kalman filtering methods. Tremor activities were applied to detect Parkinson’s symptoms in this method. Sleeping tremors were identified using ML approaches based on local field potentials. The data were obtained from 12 people. The Kalman filter enhanced the attributes of classified results based on the processed data [47].

For evaluating this disease, Ref. [48] examined phonation and acoustic signals. Four distinct ML approaches were used to preprocess and evaluate the data acquired about voice frequencies. Various microphone devices, including smartphones, were used to record the voice signals. For testing the measured accuracy rate and error rate in detection, the voice features acquired using smartphones were loaded into an ML system. The acoustic cardioid (AC) channel had a 94.55% accuracy, a 0.87 area under curve (AUC), and a 19.01% equal error rate (EER). While, by using the smartphone channel, they achieved an accuracy of 92.94%, an AUC of 0.92, and EER of 14.15%, respectively.

Using EEG signals recorded during the completion of verbal fluency tests, Almalaq et al. [49] explored the connections and causality of distinct areas of the brain. Mental demands, such as transitioning between one behavioral task and another, are challenging for those with the subjective disease. Motor and phonemic fluency are among the behavioral tasks. Their approach included verbal generating skills, as well as stimulating several Broca sections of the Brodmann areas (BA44 and BA45).

In [50], the authors presented a neural network (NN) approach for identifying symptoms of the subjective disease using speech data. The algorithm helped to classify symptoms of this disease and balance the data features using the SMOTE algorithm. Furthermore, the techniques of ensemble and Adaboost were used to improve the disease detection rate (accuracy rate). The final AdaBoost ensemble classifier implementation of NNge achieved an accuracy rate of 96.30%.

The authors examined PD subject detection using various ML techniques [51]. They conducted their experiment on both training and test data, where they used 22 acoustic features of 195 sound recordings. To diagnose PD, four machine learning classifiers were used: KNN, SVM, Naive Bayes, and random forest. The Naive Bayes algorithm diagnosed PD patients with 70.26% accuracy and a precision of 0.64 for test data.

In [52], the authors proposed a method to diagnose PD using the selection and extraction of features and pre-processing classification. In their work, for the feature selection task, recursive feature elimination and feature importance methods were used. For classification, various ML algorithms were used, such as SVM, ANN, and Classification and Regression Trees (CART). The accuracy of classification was measured before and after feature selection. Before feature selection, SVM was shown to have 79.98% accuracy, and after selection, it was shown to implement better than that.

The authors proposed a statistical method to detect the subjective disease using voice features including vowels. They used two ML techniques, SVM and KNN, where the accuracy rates obtained were 91.25% and 91.23%, respectively [53].

In [54], the authors suggested a method for evaluating feature sets by comparing performance metrics with various feature sets, such as genetic algorithm-based feature sets and Principal Component Analysis (PCA)-based feature reduction techniques. Using SVM with RBF and genetic algorithm-based feature sets, they were able to achieve an accuracy of 97.57%.

Using L1-norm SVM of feature selection, Ref. [55] suggested a method for identifying PD patients from healthy people by generating a new subset of features from the PD dataset. Their study was validated using the k-fold cross-validation approach. The results of their study’s experiments imply that the suggested approach may be used to reliably forecast the subjective disease and that it can be readily used in healthcare for diagnosis purposes.

According to [56], Linear Discriminant Analysis (LDA) performed better than PCA for distinguishing HC subjects and PD patients; thus, LDA was used as input for the clustering models. The performance of various models was evaluated by comparing the results of the clustering algorithms with the ground truth after a follow-up. In terms of sensitivity, specificity, and accuracy, Hierarchical clustering surpassed DBSCAN and K-means algorithms by 78.13%, 38.89%, and 64%, respectively.

From the review above, it was observed that various ML techniques have been applied in recent research works over voice-based PD detection and in handwritten patterns to diagnose PD.

Table 1 illustrates the review of ML techniques used to diagnose one of the major symptoms of PD, speech recording, where data were collected from the UCI machine learning repository and the University of Oxford (UO) for 20 studies. For diagnosis of the subjective disease, various ML algorithms were used, such as linear kernel SVM, ANN, KNN, naïve Bayes, logistic regression, CART, random forest, etc. For these studies, the tools that were used are Weka, Matlab, OpenCV-2.49, R Programming, and Python. It was observed from Table 1 that the highest accuracy to diagnose PD was obtained using L1-Norm SVM with K-fold cross-validation; K = 10 having a 99% accuracy rate [55]. The minimum accuracy was obtained from naïve Bayes, with an accuracy rate of 70.26% [51].

Table 2 represents the review of ML approaches in handwritten patterns to diagnose PD; data were collected from the UCI machine learning repository, Parkinson’s Progression Markers Initiative (PPMI) database, Parkinson’s disease Handwriting (PaHaW) database, and the Picture Archiving and Communication System (PACS) for 20 studies. For this study, various algorithms were used such as multilayer perceptron, logistic regression, random forest, optimum path forest, ensemble AdaBoost, SVM, Soft margin multiple kernel learning, KNN, ANN, etc. The maximum number of subjects considered in Table 2 is 961, and these subjects were gathered from PPMI and local. A total of 657 subjects were collected from PPMI, of which 448 were considered PD patients and 209 were considered healthy controls, whereas 304 subjects were collected from local, of which 191 were PD patients and 113 were healthy controls [66]. For these datasets, the outcomes were obtained using the SVM algorithm. For local data, the accuracy ranged between 88 and 92%, and for PPMI, it ranged from 95 to 97%. All the experiments were conducted using different tools, i.e., Weka, Matlab, Python, etc. It was observed that the best accuracy was obtained using SVM (linear kernel) with an accuracy rate of 97.9% [67], having 652 subjects with 443 PD patients and 209 healthy controls. The minimum accuracy was obtained using SVM with an accuracy rate of 78.4% for 550 subjects, of which 342 were PD patients and the rest were healthy controls.

## 5. Adaptation of the ML Framework

Multiple input variables led to various interpretations. When the input variable is an acoustic voice feature, ML algorithms are preferred to diagnose PD. In the instance of acoustic voice datasets, the primary interpretation for the application of ML was to diagnose the initial signs of PD [86]. In other instances, it was assumed that training models may be quite effective for the early screening of PD because the gold standard was readily available. A particular combination of ML methods, including PCA, was used since the input dataset’s dimensions were reduced. A decision tree or k-mean clustering algorithms are more suitable for analyzing the speech database’s characteristics, and these classifiers may be used to classify voice data for control vs. PD. Due to the fact that the acoustic speech data violated the data in components, it was considered that learning the acoustic speech data using ML techniques such as HMM would be the best approach, which would then be followed by the detection procedure. To determine the risk of PD, a deep CNN classifier using transfer learning and data augmentation approaches can be implemented. Due to the small amount of data, using handwriting data to predict PD presents a significant classification difficulty in the early stages. To achieve high accuracy, the independent usage of the ImageNet and MNIST databases as input sources was utilized.

### 5.1. Architecture Based on Acoustic Voice Dataset as Input

**Figure 5:**
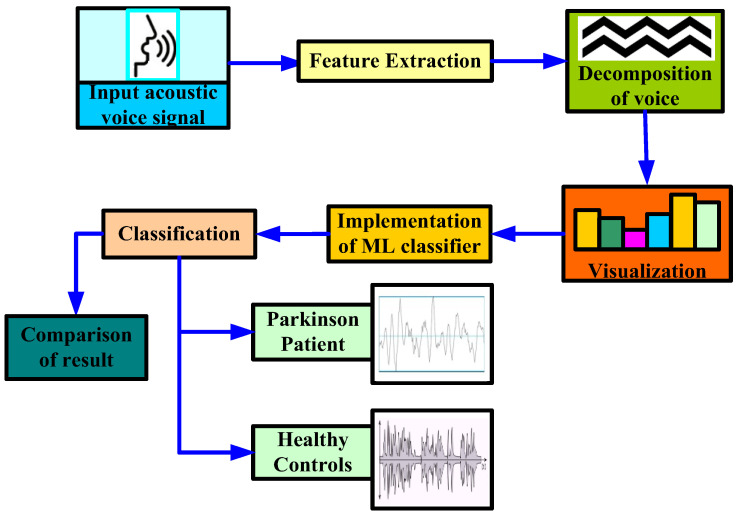
Proposed methodology to diagnose Parkinson’s disease by [87].

In [87], the authors proposed a methodology to diagnose PD using stochastic gradient descent (SGD), logistic regression, Extreme Gradient Boosting (XGB), KNN, random forest, and decision tree ML classifier, as shown in Figure 5. In their study, the authors first extracted certain attributes to classify for better understanding. By extracting attributes from the input data, feature extraction improves the accuracy of trained models. By getting rid of the redundant records, this stage decreases the dimensionality of the data. Naturally, it speeds up categorization. By choosing and merging variables into features, it helps acquire the optimum feature from such enormous data sets, while also significantly reducing the volume of data. These characteristics are simple to use while still accurately and uniquely describing the real data set. Secondly, they applied some data mining approaches to classify the HC and affected patients based on various acoustic voice features to predict the accuracy rate. For that, the authors first set the target variables, i.e., the health status of PD patients. Once the target attribute was set, they modified the dataset column that was used as the input after being extracted from the dataset. Finally, the authors made a comparison among all the ML algorithms to check the best accuracy result, which was obtained by a random forest classifier with an accuracy rate of 97.10%, and a minimum accuracy was obtained by SGD and logistic regression, with an accuracy rate of 91.66% for both classifiers.

### 5.2. Architecture Based on Handwritten Patterns as Input

**Figure 6:**
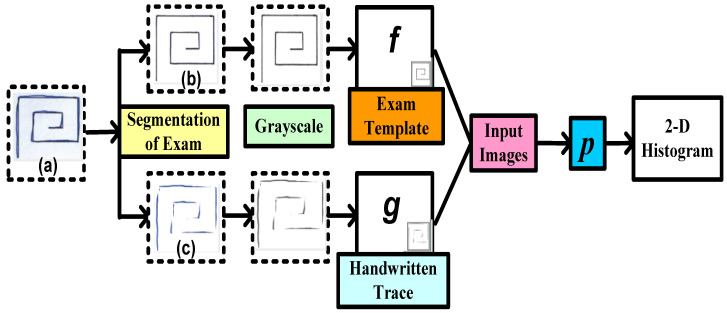
Proposed methodology to diagnose Parkinson’s disease using handwriting in a spiral format by [68].

A structural Co-occurrence Matrix (SCM)-based technique to diagnose PD as shown in Figure 4 was proposed by [68]. In their research, the features were extracted from the spiral and meander handwriting exams of the Hand PD datasets [88]. Figure 6 represents a methodology with combinations of an exam template (b) and handwritten trace (c). First, the exam segmentation is performed, and it generates two new images: an exam template (b) and a handwritten trace (c). By using digital image processing techniques on a handwritten trace, these images are produced. Secondly, the segmentation of the exam is converted into grayscale for the next level. The third phase is feature extraction from the grayscale images that have been segmented and converted. As shown in Figure 4, these images serve as the SCM’s input images.

Analyzing the connection between signals, in this example in a two-dimensional space, is conducted by feature extraction through the SCM.

For this study [68], datasets were collected from 92 individuals, of which 74 were PD patients and 18 were HC. In their proposed work, various algorithms such as SVM, naïve Bayes, and OPF classifiers were applied to the dataset. The highest accuracy was calculated by combining the handwritten trace with the handwriting in a spiral format by using SVM (85.54%).

### 5.3. Architecture Based on Gait Dataset as Input

A methodology in gait patterns to diagnose PD using ML algorithms was proposed by [89]. According to the authors, gait pattern is very eccentric for each and every human being, but there is a substantial transformation in the gait pattern of HC and PD patients. Table 3 represents the review of ML approaches in gait data to diagnose PD for 18 studies. For this study, the sources of data were the Laboratory for Gait and Neurodynamics, Neurology Outpatient Clinic at Massachusetts General Hospital, Boston, MA, USA, and the University of Michigan and were collected from the participants. The ML algorithms used to diagnose PD for gait symptoms were the least square-SVM, particle swarm optimization, fuzzy KNN, random forest, hidden Markov models, logistic regression, ANN, kernel fisher discriminant, naïve Bayes, linear discriminant analysis, etc. The maximum number of subjects considered in Table 3 is 424, with 156 PD patients and 268 healthy controls, with an accuracy rate of 85.51% using the hidden Markov model algorithm. The minimum number of subjects was 20, with 10 PD patients and 10 healthy controls with an accuracy of 91.9% using a deep convolutional neural network algorithm. For experimental setup, MATLAB R2013b and python were used. It was observed that the maximum accuracy was achieved by using the SVM algorithm with a 100% accuracy rate for the 166 subjects, where 93 were PD patients and 73 were healthy controls. The minimum accuracy was achieved by the random forest algorithm, with a 79.6% accuracy rate for the 80 subjects, where 40 were PD patients and 40 were healthy controls.

## 6. Discussion: Challenges and Recommendations

The use of AI to help detect and treat diseases is of increasing interest to researchers and clinicians. Mobile technologies such as smartphones and widely used low-cost sensors produce large amounts of health data. These data may be used by AI to provide previously unattainable insights on the prevalence of diseases and patient status in a setting where people are free to move about and, furthermore, from clinical datasets. The use of AI can help with global epidemiology initiatives and patient symptom monitoring. Despite how stimulating these applications are, it is important to consider both the value and potential limitations of these cutting-edge analytical techniques. The most promising applications of AI are yet futuristic. Future applications of AI to relevant datasets will, for instance, help in characterizing the molecular subtypes of Parkinson’s disease. This will make it possible for doctors to pair their patients with appropriate molecular treatments, which will advance precision medicine. Prioritizing customized monitoring, AI will continue to be most useful until it can demonstrate its ability to advance the field of precision medicine [109]. We compiled the remaining drawbacks and challenges and we suggested possible future recommendations that might result in effective ML techniques to solve the problems.

### 6.1. Challenges

Although the predicted accuracy attained by various ML techniques proposed in various automated PD detection studies was good, the adoption of the ML framework with cloud computing and edge computing in medical healthcare is presently not supported [110,111]. Neurologists and other medical professionals do not feel confident using these technologies to diagnose Parkinson’s disease in their current state. This is a result of several issues, which are described below:

- Manifold modeling

It has been extensively shown that PD is a multifaceted disorder; hence, PD prognosis or forecasting in its early stages have to carefully take into account multivariate data. Conventional computational techniques have a significant barrier in figuring out how to combine diverse data, including genetic, genomic, neuroimaging, clinical, social demography, and environmental exposure data. The easiest technique to manage heterogeneous data is to transform each type of data into a vector format before processing and then carefully concatenate all of the vectors unique to each subject into a large vector. This has traditionally been the most used method for PD diagnosis and phenotypic prediction.

- Model Interpretation

The interpretation of the model has long been a source of concern in machine learning, particularly in the field of medicine, where it is crucial to have both the information needed to make a choice and the performance of the model in generating predictions. For instance, while identifying biomarkers, a researcher can anticipate that a certain gene’s expression level will play a role in differentiating between PD and HCs, providing insight into the model’s choice of the gene as a biomarker or its omission. The standard machine learning models, such as Bayesian, rule-based models (decision tree and random forest), logistic regression, SVM, etc., are intuitively able to estimate feature contributions when training the models in this situation. This could be a factor in the fact that these methods are used in the majority of the examined research.

### 6.2. Recommendations

**Figure 7:**
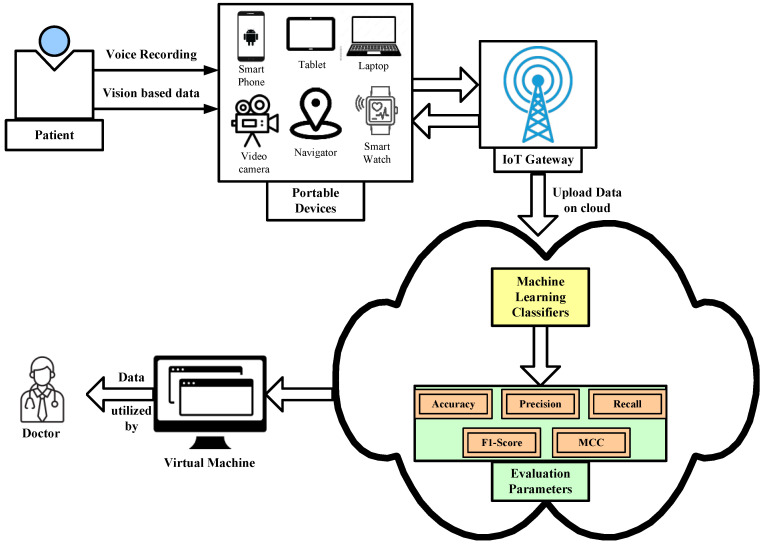
Proposed architecture of cloud and machine learning-based framework for the diagnosis of Parkinson’s disease.

We evaluated the selected articles in terms of both merits and demerits. We started our search for potential avenues for future study after considering the suggestions for critical reviews [112]. We categorized our findings that deal with identical or similar issues and defined them as follows:

- The adoption of real-time and customized based devices with an advanced computing unit is necessary to diagnosis Parkinson’s disease in real-time data through image and sensory data. It has already been proven that ML models have the capability to detect any anomalies of real-time data generated from the IoT-based devices. Edge computing can be integrated with customized devices to compute data at the edge network and provide the results at the same time.
- At present, the researchers have realized different ML models that diagnose Parkinson’s disease on the basis of individual symptoms. The researchers need to focus on developing an ML model that combinedly uses all the symptoms as input parameters for Parkinson’s disease. A light-weight portable device can be used to diagnose the various symptoms of PD by measuring several parameters such as accuracy, precision, sensitivity, recall, etc. This device should be easily wearable and washable, and it should be able to identify the different stages of the disease, along with analyze the changes due to medication treatment.
- Currently wearable sensors are limited to the diagnosis of Parkinson’s disease on the basis of gait parameters. There is a need for embedding the other modules in the wearable device that is capable of detecting Parkinson’s disease. Researchers need to focus on developing wearable sensor devices not only for one symptom but also for diagnosing the other symptoms as well. For instance, a wrist-worn device may be developed and it may be able to collect data continuously over a long period of time and identify different PD symptoms.
- Cloud- and ML-based frame works diagnose Parkinson’s disease by analyzing individual speech disorders, handwriting parameters, and many more symptoms of the subjective disease based on a cloud computing platform. Here, a patient’s file will be stored in the cloud database where patients can give their sample in the form of a voice recording based through a portable device as shown in Figure 7. The data will then be uploaded in the cloud platform for analysis and classification by using different ML classifier models. Once the patients’ data (based on various symptoms) are diagnosed by the ML classifiers in the cloud platform, the system will automatically generate a decision on whether the patient has symptoms related to PD or not. If the patient’s sample is positive based on PD symptoms, then the system will directly send information to the concerned physician. Once the physician checks all the reports, he will then upload his advice and recommendations to the cloud platform, and patients can easily receive them by their portable device.

- As future work, we intend to study the utilization of big data analytics tools along with AI approaches to diagnose more severe infections and control their spread in a timely manner.

During the survey of the research articles, the major problem we faced was the imbalanced dataset. We observed that, when applied to two distinct datasets, the same classification model can perform completely differently. Refs. [9,12] asserted this, using the example of vocal disability that sustained vowel function better than any other voice patterns. The reading task yields the highest performance; the skewed dataset in the two studies is the cause. As much data collecting as is feasible would be one option, but there are still issues with large-scale data collection.

## 7. Conclusions

Managing PD in day-to-day life is very challenging for an individual. Therefore, a good screening procedure will be beneficial, especially in circumstances where a physician’s treatment is not necessary. Thus, for the diagnosis of PD, ML algorithms were evaluated. The main aim of this review was to identify existing ML-based research to diagnose PD in terms of handwritten patterns, voice attributes, and gait dataset and to determine the most appropriate technique to diagnose the PD with an accuracy rate. From the review, it was observed that the best accuracy for voice features to diagnose PD was obtained by L1-Norm SVM with K- fold cross-validation, with 99%; in handwritten patterns, it was obtained by bagging ensemble, with 97.96%; and for gait analysis, it was obtained by SVM with 100%. This review addressed various challenges and also provided some future recommendations and opportunities, as we observed that there is still a lot of work that has to be performed in the future. This review is also meaningful for the developments in neural networks and related learning systems, which provide valuable insights and guidelines for future progress.

## Figures and Tables

**Table 1:** Comparative studies of machine learning approaches in speech recording to diagnose PD.

Reference	Machine Learning Algorithms Used	Objective	Tools Used	Source of Data	No. of Subjects	Outcomes
Benba, A. et al., 2015 [40]	Linear kernel SVM	Classification of PD from HC	Not mentioned	Department of Neurology Cerrahpas‚ a Faculty of Medicine, Istanbul University	34, 17 PD + 17 HC	Classification Accuracy = 91.17%
Mathur, R. et al., 2019 [41]	ANN, KNN with K-fold cross validation; K = 10	Classification of PD from HC	Weka	UCI machine learning repository	195 instances, 24 attributes	Accuracy of: KNN with Adaboosta.M1—91.28% KNN with Bagging—90.76% KNN with MLP—91.28%
Sakar et al., 2019 [44]	Naïve Bayes, Logistic regression, SVM (RBF and Linear), KNN, random Forest, MLP	Classification of PD from HC	JupyterLab with python programming language	Collected from participants	252, 188 PD + 64 HC	Highest accuracy obtained from SVM (RBF)—86%
Yasar, A. et al., 2019 [45]	Artificial Neural Network	Classification of PD from HC	MATLAB	Collected from participants	80, 40 PD + 40 HC	Accuracy of ANN—94.93%
Almeida, J.S. et al., 2019 [48]	KNN, MLP, Optimum Path Forest (OPF), SVM with RBF, Linear and Polynomial kernel	Classification of PD from HC	OpenCV-2.49	UCI machine learning repository	98, 63 PD + 35 HC	acoustic cardioid (AC) accuracy—94.55%
Alqahtani, E.J. et al., 2018 [50]	NNge and ensemble algorithm, AdaBoostM1 with 10- fold cross validation	Classification of PD from HC	Weka	Collected from participants	31, 23 PD + 8 HC	Accuracy—96.30%
Avuçlu, E., Elen, A., 2020 [51]	KNN, random forest, naïve Bayes, SVM	Classification of PD from HC	JupyterLab with python programming language	UCI machine learning repository	31, 23 PD + 8 HC	Highest accuracy achieved from SVM—88.72% and lowest accuracy from naïve Bayes—70.26%
Zehra Karapinar, 2020 [52]	CART, ANN, SVM	Classification of PD from HC	Weka	Collected from participants	31, 23 PD + 8 HC	Highest accuracy from SVM—93.84%
Yaman, O. et al., 2019 [53]	SVM, KNN	Classification of PD from HC	MATLAB	Collected from participants	31, 23 PD + 8 HC	Accuracy rate of SVM—91.25% and KNN—91.23%
Aich, S. et al., 2019 [54]	Random forest, Bagging CART, SVM, Boosted C5.0	Classification of PD from HC	Not mentioned	Collected from participants	31, 23 PD + 8 HC	Highest accuracy obtained from SVM with RBF kernel—97.57%
Haq, A.U. et al., 2019 [55]	L1-Norm SVM with K- fold cross validation; K = 10	Classification of PD from HC	Python	University of Oxford (UO)	31, 23 PD + 8 HC	Accuracy rate—99%
Wu et al., 2017 [57]	Generalized Logistic Regression Analysis (GLRA), SVM, Bagging ensemble	Classification of PD from HC	Not mentioned	Collected from participants	31, 23 PD + 8 Healthy control (HC)	Optimal result obtained from bagging ensemble; sensitivity—97.96%, specificity—68.75%
Peker, 2016 [58]	SVM with RBF kernel	Classification of PD from HC	Weka	University of Oxford (UO)	31, 23 PD + 8 HC	Accuracy—98.95%
Montaña et al., 2018 [59]	SVM with k-fold cross validation; k = 10	Classification of PD from HC	Weka	UCI machine learning repository	54, 27 PD + 27 HC	Accuracy—94.4%
Kuresan et al., 2019 [60]	Hidden Markov Models (HMM), SVM	Classification of PD from HC	MATLAB	Collected from participants	40, 20 PD + 20 HC	Highest accuracy obtained from HMM with accuracy—95.16%, sensitivity—93.55%, specificity—91.67%
Marar et al., 2018 [61]	Naïve Bayes, ANN, KNN, random forest, SVM, logistic regression, decision tree (DT)	Classification of PD from HC	R programming	Collected from participants	31, 23 PD + 8 HC	Highest accuracy obtained from ANN—94.87%
Sheibani, R. et al., 2019 [62]	Ensemble-based method	Classification of PD from HC	JupyterLab with python programming language	UCI machine learning repository	31, 23 PD + 8 HC	Accuracy obtained from ensemble learning—90.6%,
Moharkan et al., 2017 [63]	KNN	Classification of PD from HC	Python	Collected from participants	31, 23 PD + 8 HC	Accuracy obtained from KNN—90%,
Sztahó, D. et al., 2019 [64]	ANN, KNN, SVM with RBF and linear kernel, DNN	Classification of PD from HC	Not mentioned	UCI machine learning repository	88, 55 PD + 33 HC	Highest accuracy obtained from SVM with RBF kernel—89.3%, sensitivity—90.2%, specificity—87.9%
Tracy, J.M. et al., 2020 [65]	Logistic regression (L2- Regularized), random forest, Gradient Boosted trees	Classification of PD from HC	Python	mPower database	2289, 246 PD + 2023 HC	Highest accuracy obtained from gradient boosted trees recall—79.7%, precision—90.1%, F1-score—83.6%

**Table 2:** Comparative studies of machine learning approaches in handwritten patterns to diagnose PD.

Reference	Machine Learning Algorithms Used	Objective	Tools Used	Source of Data	No. of Subjects	Outcomes
Taylor, J.C. and Fenner, 2017 [66]	SVM with 10-fold cross-validation	Classification of PD from HC	MATLAB	PPMI and local database	PPMI: 657, 448 PD + 209 HC and local: 304,191 PD + 113 HC	Local data: Accuracy for local data range between 88 to 92% and for PPMI range from 95 to 97%
Oliveira et al., 2017 [67]	SVM with linear kernel, logistic regression with LOOCV, KNN	Classification of PD from HC	C++ Programming language and MATLAB R2014a	PPMI database	652, 443 PD + 209 HC	SVM (linear kernel) with highest accuracy rate—97.9%
de Souza et al., 2018 [68]	OPF, naïve Bayes, SVM (RBF) with cross validation	Classification of PD from HC	Python	HandPD	92, 74 PD + 18 HC	Highest accuracy obtained from SVM with RBF kernel—85.54%
Drotár et al., 2016 [69]	SVM, KNN, Ensemble AdaBoost	Classification of PD from HC	MATLAB	PaHaW database	75, 37 PD + 38 HC	Highest Accuracy obtained from SVM—81.3% with specificity—80.9% and sensitivity—87.4%
Hsu, S.-Y. et al., 2019 [70]	SVM with RBF kernel, logistic regression	Classification of PD from HC	Weka	PACS	202, 94 Severe PD + 102 mild PD + 6 HC	Highest accuracy obtained from SVM-RBF 83.2%, having sensitivity 82.8%, specificity 100%
Khatamino et al., 2018 [71]	Convolutional Neural Network (CNN)	Classification of PD from HC	Python Programming	Collected from participants	72, 57 PD + 15 HC	Accuracy—88.89%
Kurt, İ.et al., 2019 [72]	SVM (linear and RBF kernel), KNN	Classification of PD from HC	Not mentioned	UCI machine learning repository	72, 57 PD + 15 HC	Highest accuracy obtained from SVM (linear)—97.52%.
Mabrouk et al., 2019 [73]	Random forest, SVM, MLP, KNN	Classification of PD from HC	Not mentioned	PPMI Database	550, 342 PD + 157 HC + 51 Scan without evidence of dopaminergic deficit (SWEDD)	For motor features, highest accuracy obtained from SVM—78.4%, and for non-motor features, highest accuracy obtained from KNN—82.2%
Fabian Maass et al., 2020 [74]	SVM	Classification of PD from HC	Weka	UCI machine learning repository	157, 82 PD + 68 HC +7 Normal Pressure Hydrocephalus (NPH)	Sensitivity—80%, and specificity—83%
Mucha, J. et al., 2018 [75]	Random forest classifier	Classification of PD from HC	Python Programming	PaHaW Database	69, 33 PD + 36 HC	Obtained classification accuracy—90% with sensitivity 89%, and specificity 91%
Cibulka et al., 2019 [76]	Random forest	Classification of PD from HC	Not mentioned	Collected from participants	270, 150 PD + 120 HC	Classification error for rs11240569, rs708727, rs823156 is 49.6%, 44.8%, 49.3%, respectively.
Pereira, C.R. et al., 2016 [77]	CNN with cross validation	Classification of PD from HC	Not mentioned	Collected from participants	35, 14 PD + 21 HC	Accuracy rate of CNN—87.14%
Prashanth, R. et al., 2016 [78]	Naïve Bayes, random forest SVM, boosted trees	Classification of PD from HC	MATLAB	PPMI database	584, 401 PD + 183 HC	Highest accuracy obtained from SVM with RBF kernel—96.40% having sensitivity 97.03% and specificity 95.01%
Shi, et al., 2018 [79]	Soft margin multiple kernel learning (SMMKL) with LOOCV	Classification of PD from HC	Not mentioned	PPMI database	33, 15 PD + 18 HC	Accuracy rate—84.85% with sensitivity 80% and specificity 88.89%
Trezzi, J. P et al., 2017 [80]	Logistic regression	Classification of PD from HC	Not mentioned	UCI machine learning repository	87, 44 PD + 43 HC	Sensitivity 79.7% and specificity 80%
Wenzel et al., 2019 [81]	CNN	Classification of PD from HC	MATLAB	PPMI database	645, 438 PD + 207 HC	Accuracy—97.2%
Segovia, F. et al., 2019 [82]	SVM with 10 cross validation	Classification of PD from HC	Python programming	Virgen De La Victoria Hospital, Malaga, Spain	189, 95 PD + 94 HC	Accuracy—94.25%
Memedi, M. et al., 2015 [83]	Random forest, logistic regression, MLP and non-linear SVM	Classification of PD from HC	Weka	PPMI database	75, 65 PD + 10 HC	Highest accuracy obtained from MLP—84% having sensitivity—75.7% and specificity—88.9%
Nõmm, S. et al., 2018 [84]	Random forest, decision tree, KNN, AdaBoost, SVM	Classification of PD from HC	Python programming (Scikit–Learn Library)	Collected from participants	30, 15 PD + 15 HC	Highest accuracy obtained from Random forest—91%
Challa et al., 2016 [85]	MLP, BayesNet, boosted logistic regression, random forest	Classification of PD from HC	Weka	Parkinson’s Progression Markers Initiative (PPMI) database	586, 402 PD + 184 HC	Optimal result obtained from boosted logistic regression having accuracy—97.16%

**Table 3:** Comparative studies of machine learning approaches in gait dataset to diagnose PD.

Reference	Machine Learning Algorithms Used	Objective	Tools Used	Source of Data	No. of Subjects	Outcomes
Ye, Q. et al., 2018 [90]	Least square (LS)—SVM, particle swarm optimization (PSO)	Classification of PD, ALS, HD from HC	Not mentioned	Neurology Outpatient Clinic at Massachusetts General Hospital, Boston, MA, USA [91]	64, 15 PD + 16 HC + 13 (Amyotrophic lateral sclerosis disease (ALS)) + 20 (Huntington’s disease (HD))	Accuracy to diagnose PD from HC—90.32%, accuracy to diagnose HD from HC—94.44%, accuracy to diagnose ALS from HC—93.10%
Wahid, F. et al., 2015 [92]	Random forest, SVM, kernel Fisher Discriminant (KFD)	Classification of PD from HC	MATLAB R2013b	Collected from participants	49, 23 PD + 26 HC	The accuracy obtained from random forest, SVM, and KFD was 92.6%, 80.4% and 86.2%, respectively.
Pham, T.D.and Yan, H., 2018 [93]	LS-SVM	Classification of PD from HC	MATLAB	Laboratory for Gait and Neurodynamics	166, 93 PD + 73 HC	Sensitivity—100% and specificity—100%
Y. Mittra and V. Rustagi, 2018 [94]	Logistic regression, decision tree, SVM (Linear, RBF, Poly kernel), KNN	Classification of PD from HC	Not mentioned	Collected from participants	49, 23 PD + 26 HC	Highest accuracy obtained from SVM (RBF) and random forest—90.39%
Klomsae, A. et al., 2018 [95]	Fuzzy KNN	Classification of PD, ALS, HD from HC	Not mentioned	Neurology Outpatient Clinic at Massachusetts General Hospital, Boston, MA, USA [90]	64, 15 PD + 20 HD + 13 ALS + 16 HC	Accuracy to diagnose PD from HC—96.43%, accuracy to diagnose HD from HC—97.22%, accuracy to diagnose ALS from HC—96.88%
Milica et al., 2017 [96]	SVM-RBF	Classification of PD from HC	Python	Collected from participants from Institute of Neurology CCS, School of Medicine, University of Belgrade	80, 40 PD + 40 HC	Overall accuracy from SVM-RBF—85%
Cuzzolin, F. et al., 2017 [97]	HMM	Classification of PD from HC	Not mentioned	Collected from participants	424, 156 PD + 268 HC	Accuracy—85.51%
Félix, J.P. et al., 2019 [98]	SVM, KNN, naïve Bayes, LDA, decision tree	Classification of PD from HC	MATLAB R2017a	Neurology Outpatient Clinic at Massachusetts General Hospital, Boston, MA, USA [90]	31, 15 PD + 16 HC	Highest accuracy obtained from SVM, KNN, and decision tree—96.8%
Baby, M.S. et al., 2017 [99]	ANN	Classification of PD from HC	MATLAB	Laboratory for Gait and Neurodynamics	166, 93 PD + 73 HC	Accuracy—86.75%
Andrei et al., 2019 [100]	SVM	Classification of PD from HC	Not mentioned	Laboratory for Gait and Neurodynamics	166, 93 PD + 73 HC	Accuracy—100%
Priya, S.J. et al., 2021 [101]	ANN	Classification of PD from HC	MATLAB R2018b	Laboratory for Gait and Neurodynamics	166, 93 PD + 73 HC	Accuracy—96.28%
Perumal, S.V. & Sankar, R., 2016 [102]	SVM, ANN	Classification of PD from HC	MATLAB	Laboratory for Gait and Neurodynamics	166, 93 PD + 73 HC	Average Accuracy—86.9%
Nancy, Y. et al., 2016 [103]	Q-Backpropagated time delay neural network (Q-BTDNN)	Classification of PD from HC	MATLAB 2013	Laboratory for Gait and Neurodynamics	166, 93 PD + 73 HC	Accuracy—91.49%
Oğul, et al., 2020 [104]	ANN	Classification of PD from HC	MATLAB	Laboratory for Gait and Neurodynamics	166, 93 PD + 73 HC	Classification accuracy—98.3%
Li, B. et al., 2020 [105]	Deep CNN	Classification of PD from HC	Not mentioned	Collected from participants	20, 10 PD + 10 HC	Accuracy—91.9%
Gao, C. et al., 2018 [106]	Logistic regression, random forests, SVM, XGBoost	Classification of PD from HC	Not mentioned	University of Michigan	80, 40 PD + 40 HC	Highest accuracy obtained from random forests—79.6%
Rehman et al., 2019 [107]	SVM, logistic regression	Classification of PD from HC	Python programming	Not mentioned	303, 119 PD + 184 HC	Average accuracy—97%
Natasa et al., 2020 [108]	Random forest, XGBoosting, gradient boosting, SVM(RBF), neural networks	Classification of PD from HC	Not mentioned	Collected from the participants	10 PD	Best performance obtained from SVM(RBF) with the sensitivity value 72.34%, 91.49%, 75.00% and specificity value 87.36%, 88.51% and 93.62%, for the FoG, transition and normal activity classes, respectively.
